# YAP Activation and Implications in Patients and a Mouse Model of Biliary Atresia

**DOI:** 10.3389/fped.2020.618226

**Published:** 2021-01-21

**Authors:** Chao Zheng, Jiaqian Luo, Yifan Yang, Rui Dong, Fa-Xing Yu, Shan Zheng

**Affiliations:** ^1^Department of Pediatric Surgery, Children's Hospital of Fudan University, Shanghai, China; ^2^Institute of Pediatrics, Children's Hospital of Fudan University and Institutes of Biomedical Sciences, Shanghai Medical College, Fudan University, Shanghai, China

**Keywords:** Biliary Atresia, yes-associated protein, hippo signaling pathway, liver fibrosis, Recombinant Ankyrin Repeat Domain Protein 1

## Abstract

**Background and Aim:** Biliary atresia (BA), an inflammatory destruction of the bile ducts, leads to liver fibrosis in infants and accounts for half of cases undergoing pediatric liver transplantation. Yes-associated protein (YAP), an effector of the Hippo signaling pathway, is critical in maintaining identities of bile ductal cells. Here, we evaluated the expression of YAP and YAP target genes in BA livers and a rhesus rotavirus (RRV)-induced BA mice model.

**Methods:** Liver specimens collected from 200 BA patients were compared with those of 30 non-BA patients. Model mice liver tissues were also collected. The expression of YAP and YAP target genes were measured by transfection, RNA-seq, immunohistochemistry, immunoblot, and quantitative PCR. Masson's trichrome staining and the Biliary Atresia Research Consortium (BARC) system were utilized to score liver fibrosis status.

**Results:** The expression of YAP is elevated and positively correlated with fibrosis in BA livers. Moreover, *ANKRD1*, which is identified as the target gene of YAP, is also highly expressed in BA livers. Consistent with clinical data, YAP and ANKRD1 are significantly upregulated in RRV-induced BA mouse model.

**Conclusions:** YAP expression is closely correlated with the bile duct hyperplasia and liver fibrosis, and may serve as an indicator for liver fibrosis and BA progression. This study indicates an involvement of the Hippo signaling pathway in the development of BA, and the YAP induced ANKRD1 expression may also be related to bile duct hyperplasia in BA. This provides a new direction for more in-depth exploration of the etiology and pathogenesis of biliary atresia.

## Introduction

Biliary atresia (BA) is a rare condition in which progressive fibrosis affecting both intra- and extrahepatic biliary ducts in the newborn leads to persistent neonatal obstructive cholestasis. Though BA is a relatively rare condition, it is most common in E ast Asian region, with a incidence of 1.1 and 1.51 in 10,000 livebirths in Japan (1) and Taiwan of China (2), respectively, prevalence of which is much higher than England (0.58 in 10,000) (3), France (0.54 in 10,000) (4), and Canada (0.52 in 10,000) (5). Current knowledge of the cause of BA is largely unknown. Factors that might contribute to pathogenesis of BA are embryonic dysplasia, abnormal prenatal circulation, genetic factors, environmental toxins, viral infection, and abnormal immune response. As a palliative surgery, Kasai portoenterostomy aims to reconstruct extrahepatic bile draining structure. Though Kasai portoenterostomy at younger age has a better short-term native liver survival rate (6), most BA patients ultimately require liver transplantation on account of low survival with the native liver after portoenterostomy only (7).

The Hippo pathway plays a crucial role in regulating organ size control and tissue homeostasis (8). The core Hippo pathway is a kinase cascade in which Ste20-like kinases 1/2 (MST1/2), phosphorylate Large tumor suppressor 1/2 (LATS1/2), and LATS1/2 in turn phosphorylate Yes-association protein (YAP) and Transcriptional coactivator with PDZ-binding motif (TAZ, also known as WWTR1). As transcriptional co-activators, YAP/TAZ interact with TEA domain family members (TEAD1-4) and induce expression of target genes involved in cell proliferation and stemness. In mice liver, transgenic expression of YAP promotes cell proliferation, liver overgrowth, and tumorigenesis (9, 10). Subsequent studies indicate that YAP is highly expressed in biliary epithelial cells, and *Yap* loss of function in mice lead to degeneration of biliary epithelium in mice model and alleviate hepatic fibrosis (11–13), and *Yap* deficiency in mice prevented bile duct hyperplasia induced by bile duct ligation (14). Furthermore, elevated YAP expression in livers has been observed in American BA patients (15), indicating that YAP may associate with the development of BA.

In this study, we aimed to explore the expression pattern of YAP in Chinese BA patients and a rhesus rotavirus (RRV)-induced BA mice model, as well as the correlation between fibrosis and YAP expression, and the expression of YAP target genes in BA.

## Materials and Methods

### Patients

The study enrolled patients with type III BA (*n* = 200) and choledochal cyst (*n* = 30) first treated at the Children's Hospital of Fudan University from March 2015 to March 2017. The diagnosis of BA or choledochal cyst was performed by certified pathologists. Included infants presented no associated extrahepatic anomalies. This study was approved by the Ethics Committee of the Children's Hospital of Fudan University and all participants' parents signed informed consents.

### Sample Collection and Preparation

Liver and serum samples were obtained from patients mentioned above. Liver biopsies used for qPCR analysis were taken at the time of surgical resection, snap-frozen in liquid nitrogen, and stored at −80°C until use. Formalin-fixed paraffin-embedded (FFPE) tissues were used for immunohistochemical (IHC) staining and Masson staining.

### RNA Extraction, RT-PCR, and Sequencing

Total RNA was extracted from tissue using TAKARA MiniBEST Universal RNA Extraction Kit following the manufacturer's instructions. The complementary DNA (cDNA) were synthesized using TAKARA PrimeScript™ RT Master Mix (Perfect Real Time). RT-PCR was carried out using ABI 7500 FAST instrument and TAKARA SYBR Premix Ex Taq (Tli RNaseH Plus) kit. Relative quantification data were then calculated and analyzed by the 2^−ΔΔCt^ method. Sequences of the qPCR primer pairs (in the 5′-3′ direction) are presented in the Supplementary Table 1. RNA-Seq libraries were prepared by using Illumina's TruSeq Sample Preparation Kit. Samples were on an Illumina HiSeq 2000 for 2 ^*^ 100-bp paired-end sequencing.

### IHC and Masson's Trichrome Staining

Paraffin embedded tissue specimens were sectioned, dewaxed, and rehydrated. Antigen retrieval was performed in 10 mM sodium citrate buffer (pH 6.0) at 95–100°C for 20 min. Endogenous peroxidase activity was blocked by 3% H_2_O_2_ for 30 min. Sections were then blocked in 5% BSA for 1 h and incubated with primary antibodies overnight. After extensive washing, the sections were incubated with secondary antibodies at room temperature for 1 h. DAB solution was applied and hematoxylin was used for counterstaining. The primary antibodies used in Immunochemistry were anti-YAP (CST, 1:200), anti-ANKRD1 (Santa Cruz, 1:100), and anti-CK19 (Abcam, 1:800). Collagen fiber deposition in tissue sections were visualized by Masson's trichrome staining. Staining results were quantified using Image-Pro Plus software. IOD/Area was used to quantify the protein expression in Immunohistochemistry (IOD: integrated option density; Area: total area of staining region).

### Cell Culture, Plasmids, and Transfection

Human embryonic kidney cells (HEK293A) and MA104 cells were cultured in DMEM (Hyclone) containing 10% FBS (Gibco) and 50 mg/ml penicillin/streptomycin (P/S) at 37°C under 5% CO_2_. DNA transfection were performed using PolyJet (SignaGen) following the manufacturer's instructions.

### Animals and Virus Injection

Pregnant BALB/c mice were maintained in a pathogen-free (SPF) environment with 12 h dark/light cycle. RRV were amplified in MA104 cells and the viral titers were measured by plaque assay. Newborn pups were divided into 2 groups: the BA model group received intraperitoneally injection with 25 ul of 10^6^ PFU/mL RRV; the same volume of saline was given to the control group. Mice that were not fed by their mothers or died within the first 3 days due to other reasons were not included in the analysis. All neonatal mice were daily weighed and closely observed, including fur, bare skin, and stool. Mice were sacrificed at the end of 2 weeks and the liver tissues were fixed with 10% formaldehyde or snap frozen for further analysis.

### Statistical Analysis

Unless otherwise indicated, Student's *t*-test were used in statistical analysis. Spearman's Rank Correlation analysis was employed to determine the correlation between YAP expression and liver fibrosis. The symbols ^*^, ^**^, and ^***^ in figures indicate *P* < 0.05, *P* < 0.01, and *P* < 0.001 respectively.

### Ethics and Biosafety Statements

All experiments and mice work has been approved by the ethical committee and biosafety department at Children's Hospital of Fudan University.

## Results

### Bile Duct Hyperplasia and Fibrosis in Liver Tissues of Chinese BA Patients

**Figure 1:**
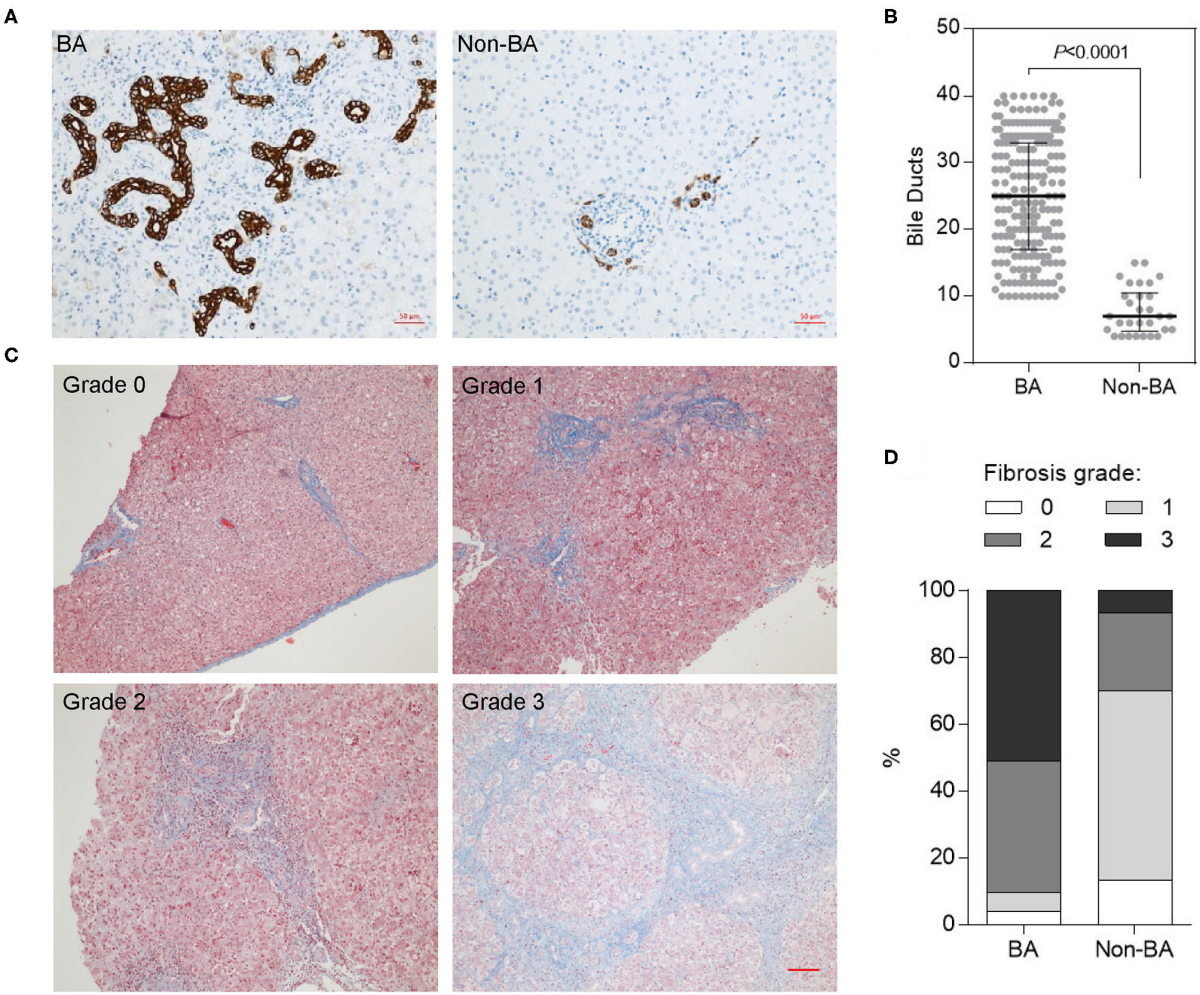
Bile duct hyperplasia and fibrosis in BA livers of Chinese patients. **(A)** Hyperplastic and deformed bile ducts in BA livers indicated by CK19 IHC staining. Scale bar: 50 μm. **(B)** Quantification of **(A)** (numbers of bile duct per field). **(C)** Fibrosis in BA livers indicated by Masson's trichrome staining. Scale bar: 100 μm. **(D)** Fibrosis grades in BA and Non-BA livers were summarized.

To investigate the molecular mechanisms underlying BA development, we collected liver biopsies from 200 Chinese BA patients (mean age 2.2 ± 0.7 months) and 30 patients with choledochal cysts (mean age 17.0 ± 4.9 months), the latter used as control samples (referred as non-BA group). Bile duct could be identified by IHC staining using biliary epithelial cell marker CK19 (16) (Figure 1A). We found that the staining of bile duct in BA livers was dramatically higher than that in control group (Figure 1B). Meanwhile, the hyperplastic bile ducts presented irregular morphology, usually with larger lumens (Figure 1A). These results indicate a phenotype of bile duct hyperplasia in BA livers.

Liver fibrosis is an important indicator of BA; therefore, we measured the degree of liver fibrosis using Masson's trichrome staining, and scored fibrosis status according to the BARC system (17) (Figure 1C). The majority BA livers were classified into grades 2 and 3, with 39.5 and 51%, respectively. However, most non-BA livers (80%) were grades 1 and 2 (Figure 1D).

### Elevated YAP Expression in Liver Tissues of BA Patients

**Figure 2:**
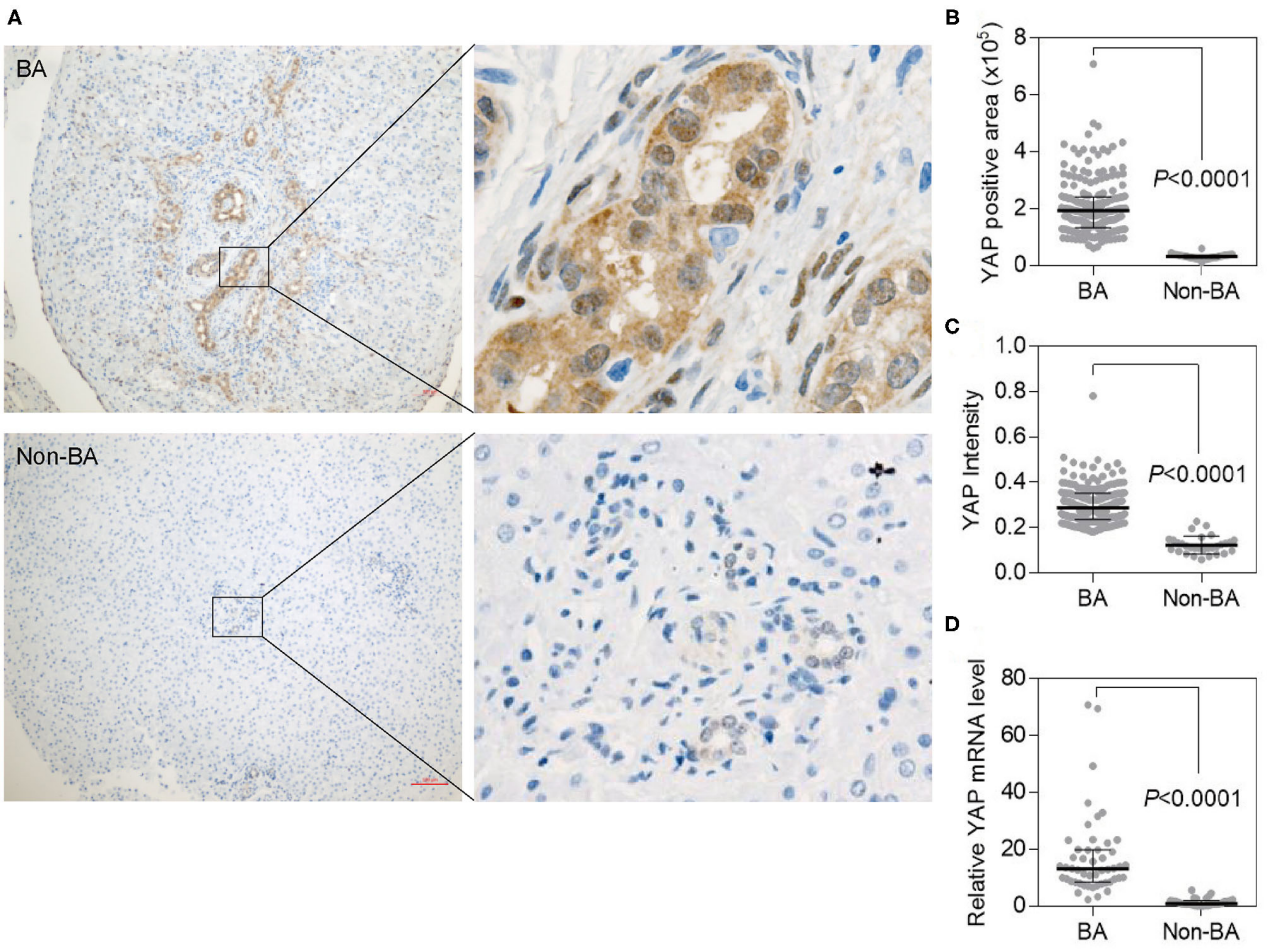
YAP expression is upregulated in BA livers. **(A)** YAP protein expression assessed by IHC staining in BA (upper panel) and non-BA (bottom panel) livers. Scale bar: 100 μm. **(B)** Quantification of YAP positive area in **(A)**. **(C)** Quantification of intensity of YAP IHC signals in **(A)**. **(D)** The mRNA level of YAP in BA and non-BA livers measured by quantitative PCR.

The expression of YAP was mainly in both nucleus and cytoplasm in biliary epithelial cells as intensity was 0.299 ± 0.081 (mean ± sd) and 0.123 ± 0.039 in BA and control livers, respectively (*p* < 0.001) (Figures 2A,C). There was no association between YAP expression with age in BA livers (Spearman test, *p* = 0.13) and Non-BA livers (Spearman test, *p* = 0.76) respectively (Supplementary Figures 2A,C). Regarding YAP positively stained area, BA and control livers had a score of 207,636 ± 95,779 and 32,057 ± 8,950 (*p* < 0.001), respectively (Figure 2B). At mRNA level, there was 13.44-fold increase in the *Yap* mRNA level in the BA group (*p* < 0.001) (Figure 2D). Thus, compared with non-BA group, YAP expression was significantly upregulated in BA livers.

### YAP Expression Is Positively Correlated With Fibrosis in BA Livers

**Figure 3:**
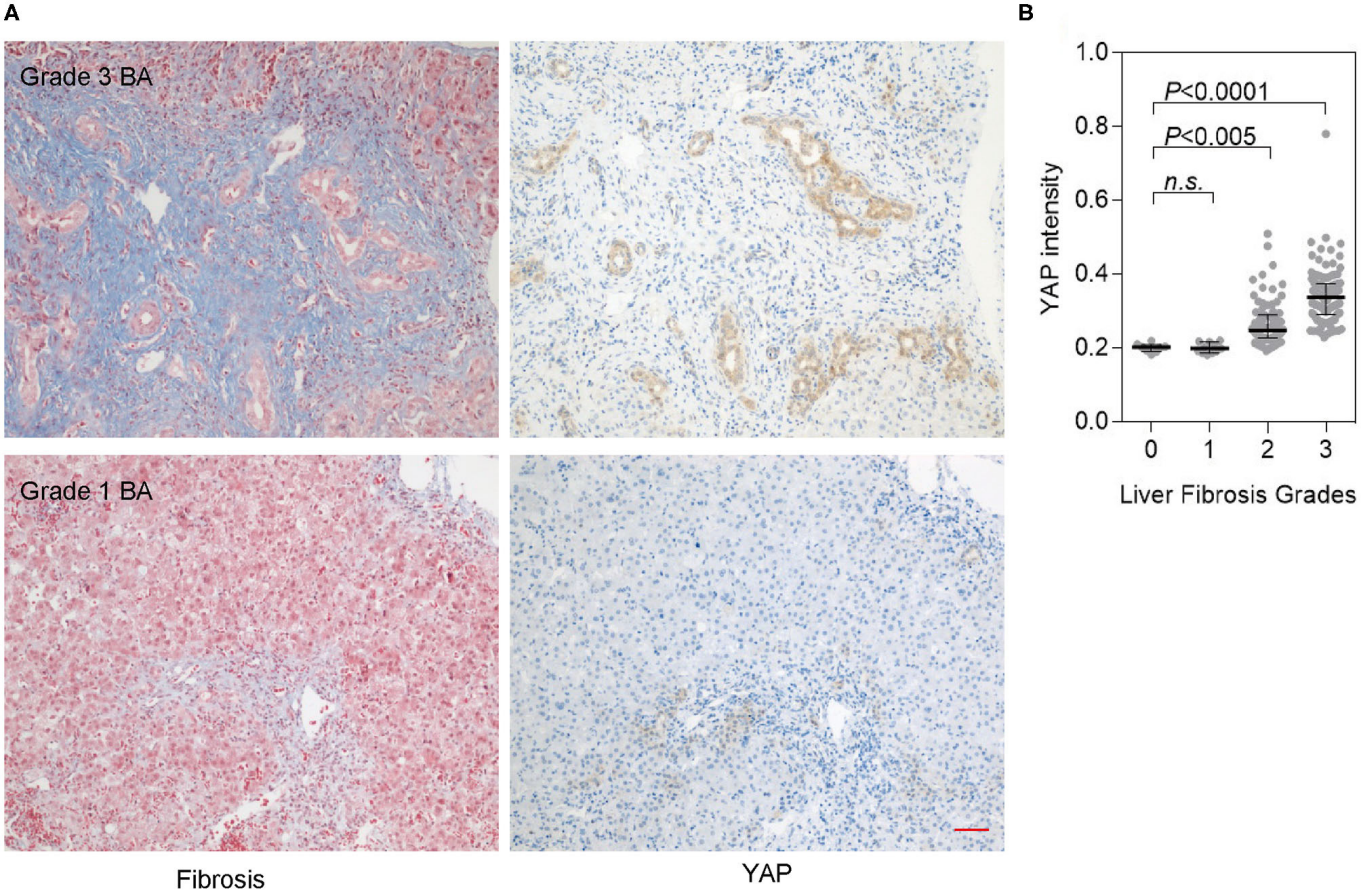
YAP expression is positively correlated with fibrosis in BA livers. **(A)** Fibrosis (Masson's trichrome staining) and YAP expression (IHC) in consecutive sections of BA livers. Scale bar: 100 μm. **(B)** Correlation between YAP expression and liver fibrosis revealed by the Spearman's rank correlation analysis. Significantly higher YAP expression was observed in Fibrosis Grade 3 and 4 BA livers.

YAP is a sensor of mechanical cues from cell microenvironment (18), and tissue fibrosis may provide a stiff matrix for cells and lead to YAP stabilization and activation. To figure out if there is a correlation between liver fibrosis and YAP activation, we compared YAP expression and fibrosis grades of different liver tissues. YAP expression was significantly increased in BA livers with grade 3 fibrosis, and further increased in BA livers with grade 4 fibrosis (Figure 3A). In a Spearman's rank correlation analysis, YAP expression was positively correlated with fibrosis score (*r* = 0.6661, *P* < 0.0001) (Figure 3B). We also analyzed YAP expression with several serum parameters reflecting liver fitness, such as alkaline phosphatase (ALP), glutamic-pyruvic transaminase (ALT), glutamic oxaloacetic, transaminase (AST), γ-glutamyl transpeptidase (GGT), direct bilirubin (DBIL), total bilirubin (TBIL), and total bile acid (TBA); however, none of these factors showed significant correlation with YAP expression in BA patients (Supplementary Table 2). Hence, liver fibrosis represents a specific factor in BA livers that is positively correlated with YAP expression, suggesting that fibrosis is an underlying mechanism for YAP activation in BA livers.

### The Expression of YAP Target Gene *ANKRD1* Is Highly Elevated in BA Livers

We established HEK293A YAP-knockout cell line through using CRISPR/Cas9. We then performed RNA-seq on YAP-knockout cell line and control cell line using the Illumina platform. Genes that exhibited more than 2-fold differentially expressed with a *P* < 0.05 were then defined as differential expressed genes (Supplementary Table 3). We compared these genes above with genes differentially expressed in BA patient livers based on published RNA-seq data (19), and we found that many YAP downstream genes, such as *ANKRD1, CTGF, AMOTL2, PALMD, ANXA3, CYR61, NEDD9, TNFRSF12A*, and *KLF5*, were highly expressed in BA livers (Supplementary Figure 1A).

**Figure 4:**
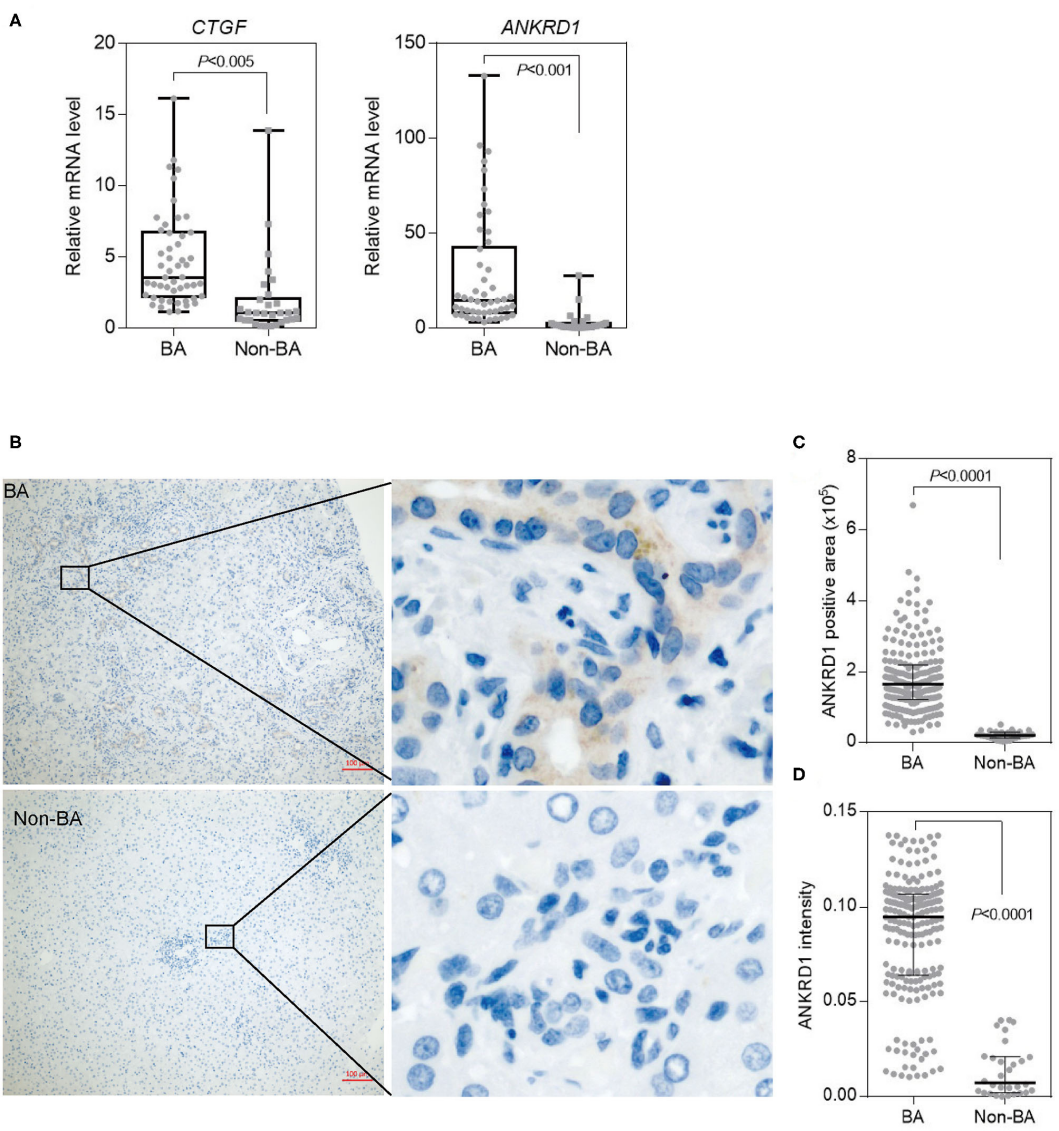
Elevated Expression of *ANKRD1* in BA livers. **(A)** Expression of YAP target genes (*CTGF* and *ANKRD1*) in BA (*n* = 50) and non-BA (*n* = 30) livers was measured by quantitative PCR. **(B)** Expression of ANKRD1 protein levels in BA and non-BA livers assessed by IHC. ANKRD1 was mainly localized at cytoplasm of biliary epithelial cells of BA livers. Scale bar: 100 μm. **(C)** Quantification of ANKRD1 positive area in **(B)**. **(D)** Quantification of intensity of ANKRD1 IHC signals in **(B)**.

In order to confirm the expression pattern of YAP downstream genes, we tested the mRNA level of *ANKRD1, CTGF, AMOTL2, PALMD, ANXA3, CYR61, NEDD9, TNFRSF12A*, and *KLF5* in more liver samples from BA (*n* = 50) and non-BA patients (*n* = 30) by quantitative PCR (qPCR). However, only two genes were significantly upregulated in BA livers (Figure 4A), in which mRNA levels of *ANKRD1* and *CTGF* were 10 times or 2.5 times higher than that in control samples (*P* = 0.0001). By IHC, we confirmed ANKRD1 expression in BA livers with a cytoplasmic localization in biliary epithelial cells (*p* < 0.0001) (Figures 4B–D). There was no association between ANKRD1 expression with age in BA livers (Spearman test, *p* = 0.65) and Non-BA livers (Spearman test, *p* = 0.98), respectively (Supplementary Figures 2B,D). A previous report has shown that *ANKRD1* was upregulated in a CCl4-induced liver injury mouse model, suggesting that ANKRD1 may also play a role in the development of liver fibrosis in BA (20).

### YAP and ANKRD1 Upregulation in a BA Mouse Model

**Figure 5:**
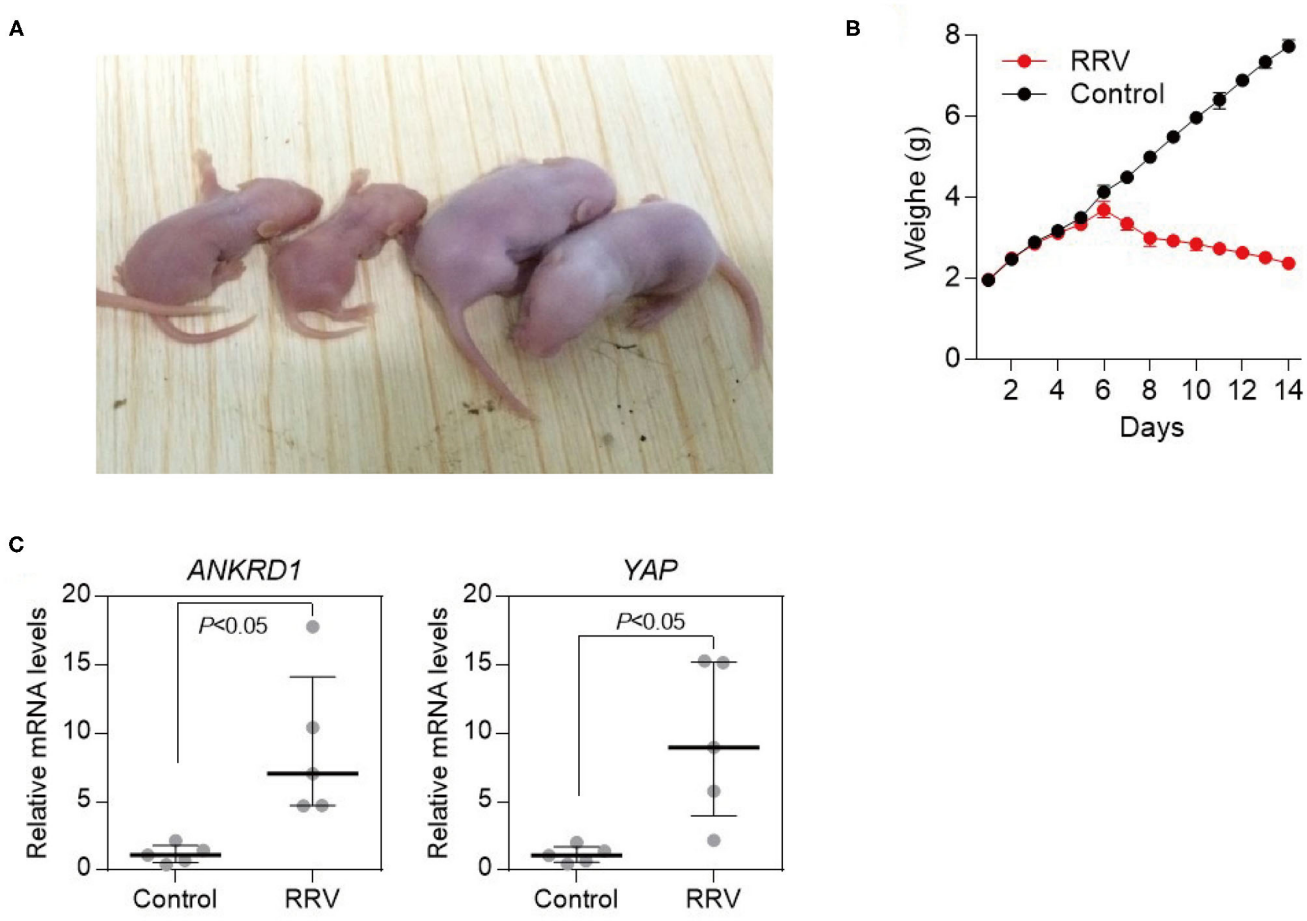
Increased YAP and ANKRD1 mRNA expression in an RRV-induced murine BA model. **(A)** RRV-induced BA model (age 2 weeks). Representative image of RRV-infected mice (left) and control (right). BA related symptoms, including reduced growth, jaundice, and oily fur were observed in RRV-infected mice. **(B)** Body weight of RRV-infected and control mice at different time points. Significant weight loss was observed in RRV-infected mice (*n* = 5). **(C)** Elevated expression of *ANKRD1* and *Yap* mRNA in RRV-induced BA mouse livers (*n* = 5).

It has been shown previously that perinatal infection of *Rhesus Monkeys Rotavirus* (RRV) of mice led to development of cholestasis and typical BA phenotypes (21). We then sought to explore the change of YAP and ANKRD1 in BA development in RRV-induced BA mouse model. The viral infected mice gradually appeared with reduced growth and body weight, as well as jaundice in non-fur-covered skin in 2 weeks (Figures 5A,B). Consistent with results of human BA livers, viral infected mice showed increased number of bile ducts with irregular lumens (*P* < 0.0001) (Supplementary Figure 1B), and more intrahepatic bile ducts were observed in the portal triads. In addition, the formation of excess fibrous connective tissue and even bridge fibrosis was observed in the RRV-infected group. According to the BARC scoring system, different grades of fibrosis were diagnosed in BA mice, whereas none of the control group developed liver fibrosis (Supplementary Figure 1C). Hence, the RRV-induced BA mouse model closely resembles BA development in infants.

**Figure 6:**
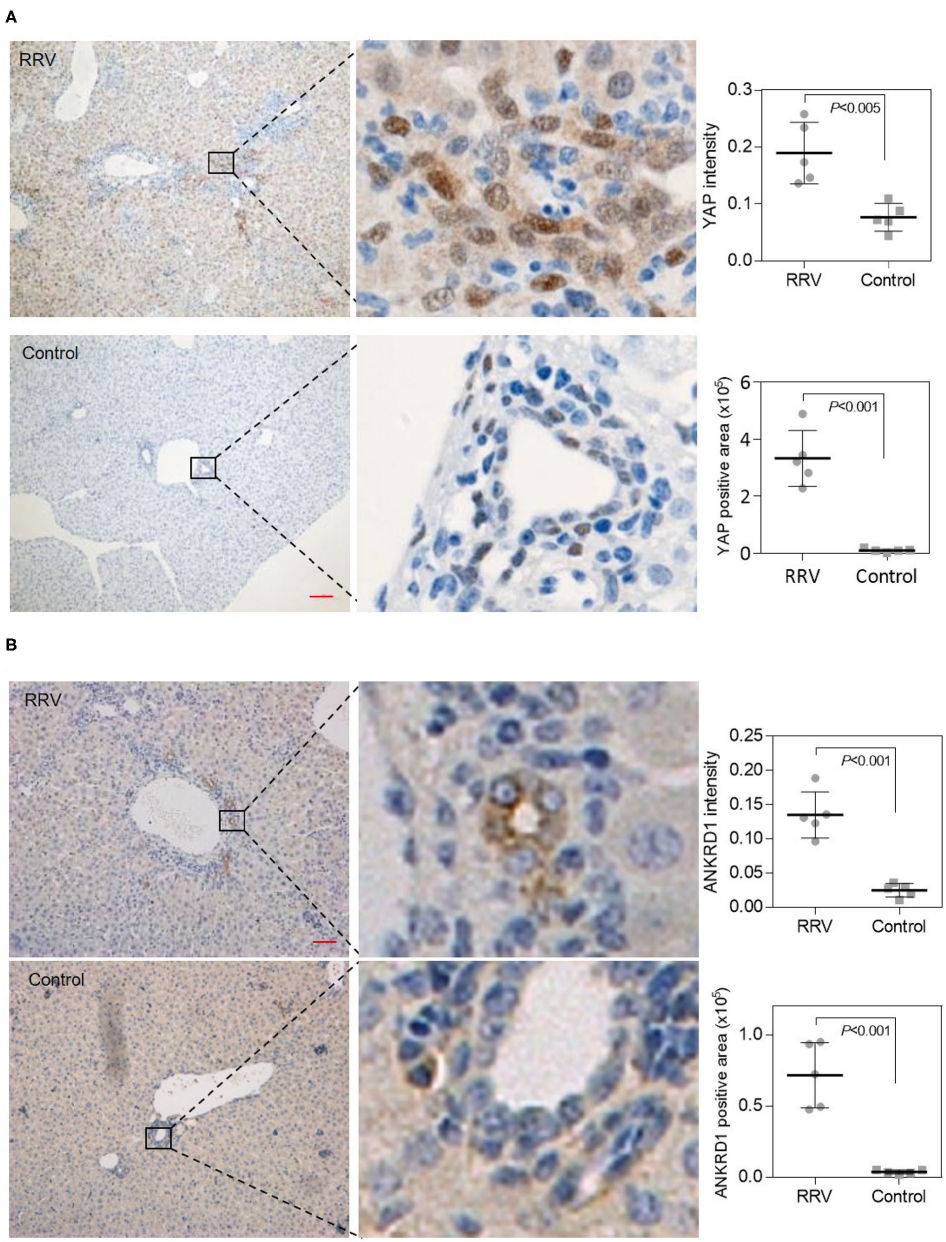
Increased YAP and ANKRD1 expression in an RRV-induced murine BA model. **(A)** Elevated YAP expression in RRV-induced BA mouse livers as assessed by IHC (left) and quantified (right). Scale bar: 100 μm. Both YAP positive area and IHC signal were increased in BA mouse livers (right) (*n* = 5). **(B)** Elevated ANKRD1 expression in RRV-induced BA mouse livers as assessed by IHC (left) and quantified (right). Scale bar: 100 μm. Both ANKRD1 positive area and IHC signal were increased in BA mouse livers (right) (*n* = 5).

By using IHC staining, we found that YAP was localized mainly in the nuclear region of biliary epithelial cells of BA mice (Figure 6A). ANKRD1 was localized at cytoplasm of biliary epithelial cells of BA mice (Figure 6B). In addition, the mRNA expression level of YAP and ANKRD1 in the liver biopsies were 8.3-fold and 7.7-fold upregulated in BA mice livers (Figure 5C). These observations indicate that, consistent with clinical data, YAP and ANKRD1 are significantly upregulated in RRV-induced BA mouse model.

## Discussion

BA is a life-threatening disease with progressive inflammation of both intrahepatic and extrahepatic biliary tracts, affecting infants in the first months after birth. Typically untreated patients succumb within the first year of life to progressive liver cirrhosis. In the last decade or so, the Hippo pathway has been shown to play a pivotal role in organ development, particularly liver (8). The key component of Hippo pathway YAP was reported to overexpress in cholangiocarcinoma and hepatocellular carcinoma (22, 23). Subsequently Bai's data suggested that YAP promoted cholangiocyte and hepatocyte proliferation after cholestatic injury in animal models. These indicated that YAP may participate in the pathogenesis of BA.

The incidence of BA is more frequent in East Asia (24), hence it is important to study if YAP is upregulated in livers of BA patients from this region. Our study illustrated that YAP expression was significantly upregulated in BA livers compared with non-BA group, which was in accordance with the result of Gurda's study (15). And the large number of samples in our study further confirmed Gurda's findings. In addition, the result that YAP expression was positively correlated with fibrosis in BA livers coincided with the study reported (ρ > 0.6, *p* ≤ 0.01) (15), either. That our research results had better consistency benefited from Image-Pro Plus software processing and statistical analysis following. Hepatic cirrhosis is an important factor in the development of BA. Here, we found that YAP expression level was tightly associated with the degree of fibrosis. Since the degree of fibrosis affects the prognosis of Kasai portoenterostomy (25, 26), YAP may also serve as a prognostic indicator for this common surgical procedure for BA. However, the mechanism of the direct pathogenic role of YAP in BA development remains unsolved. The mechanism between YAP expression and hepatic fibrosis is difficult to dissect, since YAP could be activated and stabilized by a stiff niche, and YAP activation may also lead to a stiff microenvironment by inducing expression of cell matrix-related proteins (27). Thus, fibrosis and YAP may form a positive feedback loop to facilitate BA development.

Identification of biomarkers reflexing disease status of BA is critical for decision making in the clinic. By comparing RNA-seq data from clinical BA samples and YAP knock-out cell line, we identified several common differentially expressed genes (*ANKRD1, CTGF, AMOTL2, PALMD, ANXA3, CYR61, NEDD9, TNFRSF12A*, and *KLF5*). These genes were directly or indirectly regulated by YAP proved by recent researches (28, 29). Through secondary expansive verification, we obtained two differentially expressed genes ANKRD1 and CTGF from these genes. High levels of CTGF and mRNA expression were observed in BA livers in 2005 (30, 31), and the expression was correlated with fibrosis in BA livers. This was consistent with the results of our research, and also confirmed that YAP was positively correlated with the severity of liver fibrosis.

ANKRD1, also known as Cardiac adriamycin-responsive protein, is a well-known transcription factor downstream of YAP. Different from CTGF, which is widely expression in many organs, *ANKRD1* usually specifically expresses in cardiac and skeletal muscle. Therefore, the ectopic expression of *ANKRD1* in liver may imply its pathogenetic role in hepatopathy. By IHC, we confirmed ANKRD1 expression in BA livers and RRV-infected mouse livers for the first time. A previous report has shown that *ANKRD1* was upregulated in a CCl4-induced liver injury mouse model, suggesting that ANKRD1 may also play a role in the development of liver fibrosis in BA (20). It has been shown previously that *ANKRD1* expression is increased in hepatic stellate cells during liver regeneration (20) and upon Hepatitis C Virus infection (32, 33), suggesting a role of ANKRD1 in liver function under stress, while it is unclear how this muscle specific gene is turned on in liver diseases.

In summary, the expression of YAP is increased in livers of BA patients and BA murine models. YAP expression is closely correlated with the bile duct hyperplasia and liver fibrosis. This study underscores the involvement of the Hippo signaling pathway in the development of BA, and the increased expression of YAP causing the up-regulation of downstream ANKRD1, which may be related to bile duct hyperplasia in BA. This provides a new direction for more in-depth exploration of the etiology and pathogenesis of biliary atresia.

## Data Availability Statement

The datasets presented in this study are deposited at Genome Sequence Archive for Human with accession number HRA000496, and can be accessed at: https://bigd.big.ac.cn/gsa-human/browse/HRA000496.

## Ethics Statement

The studies involving human participants were reviewed and approved by Ethics Committee of the Children's Hospital of Fudan University. Written informed consent to participate in this study was provided by the participants' legal guardian/next of kin. The animal study was reviewed and approved by Animal Ethics Committee of the Children's Hospital of Fudan University. Written informed consent was obtained from the individual(s), and minor(s)' legal guardian/next of kin, for the publication of any potentially identifiable images or data included in this article.

## Author Contributions

CZ, JL, and YY performed experiments and analyzed data. RD, F-XY, and SZ conceived, designed, and supervised this study, and wrote the manuscript. All authors approved the submitted version.

## Conflict of Interest

The authors declare that the research was conducted in the absence of any commercial or financial relationships that could be construed as a potential conflict of interest.

## Supplementary Material

Supplementary Figure 1 **(A)** Genes common differentially expressed in BA patient livers and YAP knock-out cell line compared with controls. **(B)** Bile duct hyperplasia and dysmorphia in RRV-induced BA mice livers. Bile ducts were indicated by CK19 IHC staining (left; Scale bar: 100 μm), numbers of bile ducts per portal area were quantified (right) (*n* = 5). **(C)** Fibrosis in RRV-induced BA mice livers. Fibrosis was indicated by Masson's trichrome staining (left; Scale bar: 100 μm), and fibrosis grades were assessed according to the BARC system (right) (*n* = 5).

Supplementary Figure 2 **(A)** Correlation between YAP expression and age in BA livers. There was no association between YAP expression with age (*p* = 0.13 by Spearman test). **(B)** Correlation between ANKRD1 expression and age in BA livers. There was no association between ANKRD1 expression with age (*p* = 0.65 by Spearman test). **(C)** Correlation between YAP expression and age in Non-BA livers. There was no association between YAP expression with age (*p* = 0.76 by Spearman test). **(D)** Correlation between ANKRD1 expression and age in Non-BA livers. There was no association between ANKRD1 expression with age (*p* = 0.98 by Spearman test).

Supplementary Table 1 Primer sequences used for qPCR in this study were listed as follows (in the 5′-3′ direction).

Supplementary Table 2 Correlation between liver injury serum parameters and YAP expression in BA patients.
