# Supplementary figures and images for: YAP Activation and Implications in Patients and a Mouse Model of Biliary Atresia
Source: Front Pediatr. 2021 Jan 21;8:618226. doi: 10.3389/fped.2020.618226 (PMC7859521; doi:10.3389/fped.2020.618226)

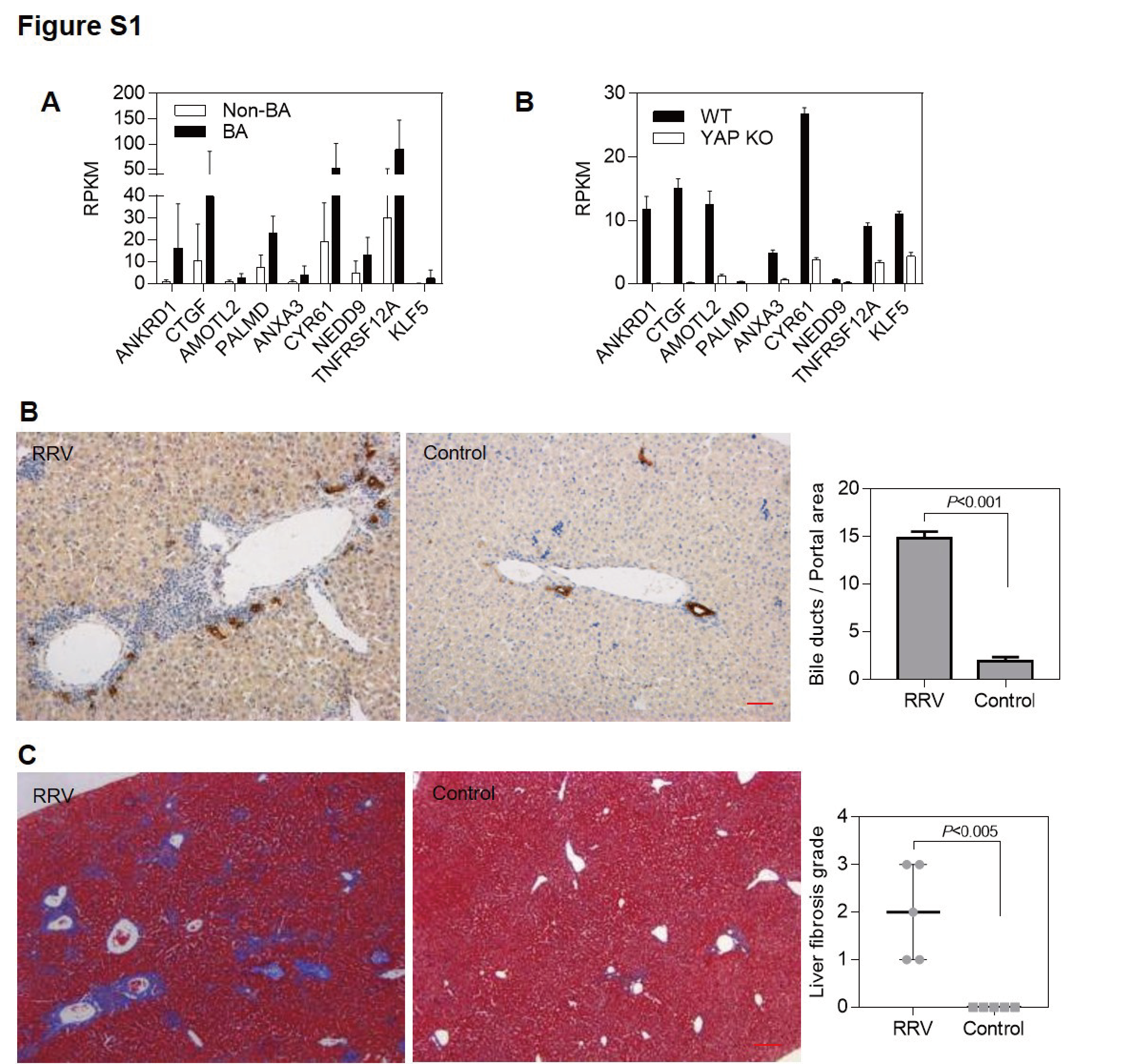

Supplement: Supplementary Figure 1 — (A) Genes common differentially expressed in BA patient livers and YAP knock-out cell line compared with controls. (B) Bile duct hyperplasia and dysmorphia in RRV-induced BA mice livers. Bile ducts were indicated by CK19 IHC staining (left; Scale bar: 100 μm), numbers of bile ducts per portal area were quantified (right) (n = 5). (C) Fibrosis in RRV-induced BA mice livers. Fibrosis was indicated by Masson's trichrome staining (left; Scale bar: 100 μm), and fibrosis grades were assessed according to the BARC system (right) (n = 5). [file Image_1.TIF]

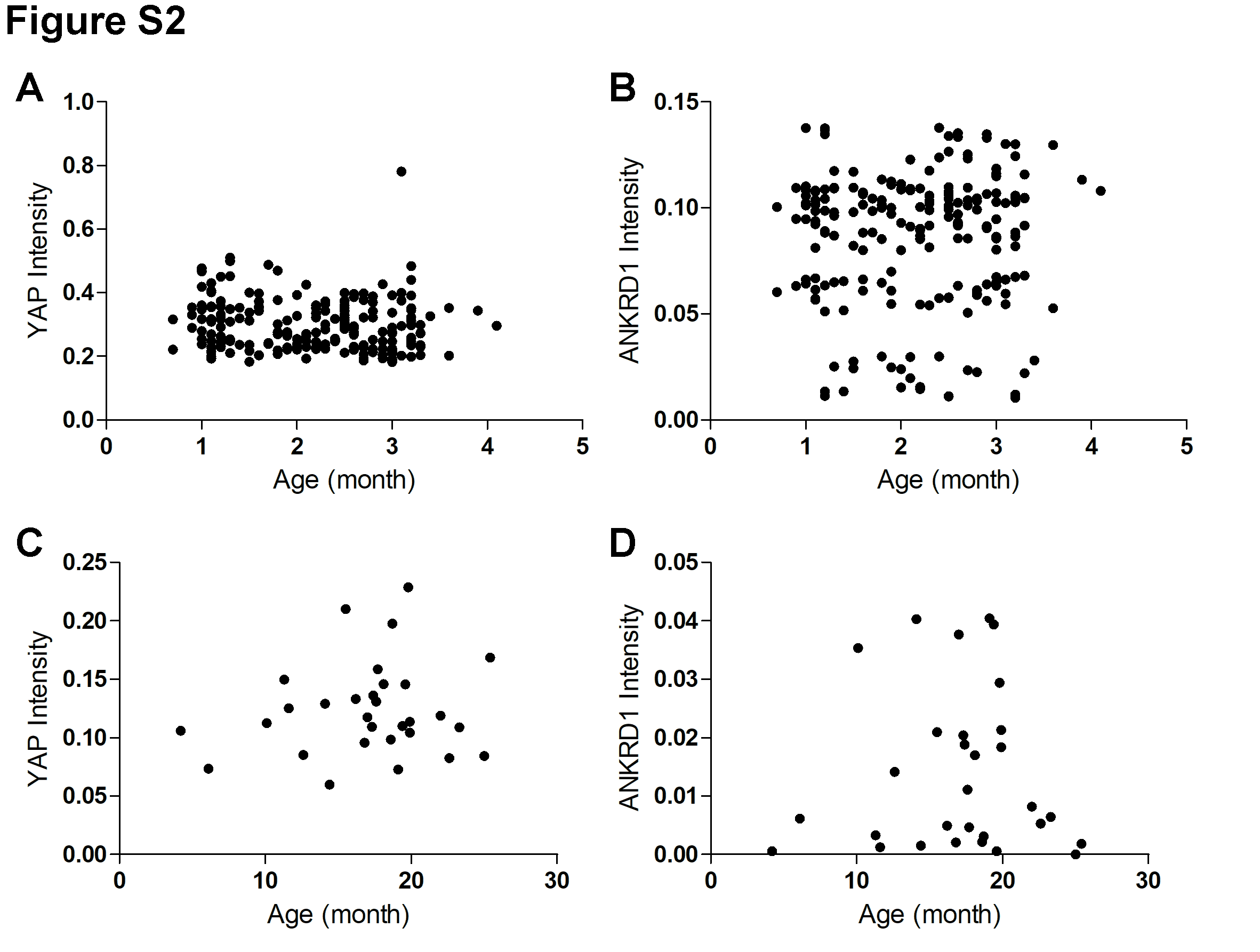

Supplement: Supplementary Figure 2 — (A) Correlation between YAP expression and age in BA livers. There was no association between YAP expression with age (p = 0.13 by Spearman test). (B) Correlation between ANKRD1 expression and age in BA livers. There was no association between ANKRD1 expression with age (p = 0.65 by Spearman test). (C) Correlation between YAP expression and age in Non-BA livers. There was no association between YAP expression with age (p = 0.76 by Spearman test). (D) Correlation between ANKRD1 expression and age in Non-BA livers. There was no association between ANKRD1 expression with age (p = 0.98 by Spearman test). [file Image_2.TIF]
