# Supplementary material for: YAP Activation and Implications in Patients and a Mouse Model of Biliary Atresia
Source: Front Pediatr. 2021 Jan 21;8:618226. doi: 10.3389/fped.2020.618226 (PMC7859521; doi:10.3389/fped.2020.618226)
Supplement: Supplementary Table 1 — Primer sequences used for qPCR in this study were listed as follows (in the 5′-3′ direction). [file Data_Sheet_1.docx]

**Supplemental Table 1. Primer sequences used for qPCR in this study were listed as follows (in the 5′-3′ direction).**

| Gene | Species | Forward primer sequence | Reverse primer sequence |
| --- | --- | --- | --- |
| ANKRD1 | human | AAGCAGGAGGATCTGAAGACACTT | GTTGTTTCTCGCTTTTCCACTGT |
| CTGF | human | AAAAGTGCATCCGTACTCCCA | CCGTCGGTACATACTCCACAG |
| AMOTL2 | human | ACCATGCGGAACAAGATGGAC | GGCGGCGATTTGCAGATTC |
| PALMD | human | TGAGGATCCATCCTTAACAGC | GGTGGTACAACTCTTAGATCACCTT |
| ANXA3 | human | CCCATCAGTGGATGCTGAAG | TCACTAGGGCCACCATGAGA |
| CYR61 | human | ACCGCTCTGAAGGGGATCT | ACTGATGTTTACAGTTGGGCTG |
| NEDD9 | human | CGTGGGTAAAAAGGTGTTCC | CAAGCCTCCAAACTCAGGAC |
| TNFRSF12A | human | AACAGAAAGGGAGCCTCACG | GTGGGGCCTAGTGTCAAGTC |
| KLF5 | human | ACACCAGACCGCAGCTCCA | TCCATTGCTGCTGTCTGATTTGTAG |
| YAP | human | TAGCCCTGCGTAGCCAGTTA | TCATGCTTAGTCCACTGTCTGT |
| Yap | mouse | ACCCTCGTTTTGCCATGAAC | TTGTTTCAACCGCAGTCTCTC |
| Ankrd1 | mouse | GGATGTGCCGAGGTTTCTGAA | GTCCGTTTATACTCATCGCAGAC |

**Supplemental Table 2. Correlation between liver injury serum parameters and YAP expression in BA patients.** Correlation coefficient (r), 95% confidence interval and p value shown for each serum parameters were calculated by Pearson analysis. Abbreviations: alkaline phosphatase (ALP); glutamic-pyruvic transaminase (ALT); glutamic oxaloacetic transaminase (AST); γ-glutamyl transpeptidase (GGT); direct bilirubin (DBIL); total bilirubin (TBIL); total bile acid (TBA).

|  | **Correlation with YAP expression (Pearson analysis)** | | |
| --- | --- | --- | --- |
|  | **r** | **95% CI** | ***P*** |
| **ALP** | 0.1810 | -0.04181 ~ 0.3867 | 0.1104 |
| **ALT** | -0.0941 | -0.3089 ~ 0.1297 | 0.4091 |
| **AST** | 0.0496 | -0.1734 ~ 0.2679 | 0.6637 |
| **GGT** | -0.0279 | -0.2475 ~ 0.1945 | 0.8072 |
| **DBIL** | 0.0134 | -0.2083 ~ 0.2339 | 0.9063 |
| **TBIL** | -0.0078 | -0.2286 ~ 0.2137 | 0.9453 |
| **TBA** | -0.1786 | -0.3846 ~ 0.04433 | 0.1153 |
